# Biogas Biocatalysis: Methanotrophic Bacterial Cultivation, Metabolite Profiling, and Bioconversion to Lactic Acid

**DOI:** 10.3389/fmicb.2018.02610

**Published:** 2018-10-31

**Authors:** Calvin A. Henard, Tyler G. Franklin, Batool Youhenna, Sergey But, Danny Alexander, Marina G. Kalyuzhnaya, Michael T. Guarnieri

**Affiliations:** ^1^National Renewable Energy Laboratory, National Bioenergy Center, Golden, CO, United States; ^2^Biology Department, San Diego State University, San Diego, CA, United States; ^3^Skryabin Institute of Biochemistry and Physiology of Microorganisms, Moscow, Russia; ^4^Metabolon, Inc., Durham, NC, United States

**Keywords:** methane, methanotroph, biogas, anaerobic digestion, lactic acid, methane biocatalysis, biogas upgrading

## Abstract

Anaerobic digestion (AD) of waste substrates, and renewable biomass and crop residues offers a means to generate energy-rich biogas. However, at present, AD-derived biogas is primarily flared or used for combined heat and power (CHP), in part due to inefficient gas-to-liquid conversion technologies. Methanotrophic bacteria are capable of utilizing methane as a sole carbon and energy source, offering promising potential for biological gas-to-liquid conversion of AD-derived biogas. Here, we report cultivation of three phylogenetically diverse methanotrophic bacteria on biogas streams derived from AD of a series of energy crop residues. Strains maintained comparable central metabolic activity and displayed minimal growth inhibition when cultivated under batch configuration on AD biogas streams relative to pure methane, although metabolite analysis suggested biogas streams increase cellular oxidative stress. In contrast to batch cultivation, growth arrest was observed under continuous cultivation configuration, concurrent with increased biosynthesis and excretion of lactate. We examined the potential for enhanced lactate production via the employ of a pyruvate dehydrogenase mutant strain, ultimately achieving 0.027 g lactate/g DCW/h, the highest reported lactate specific productivity from biogas to date.

## Introduction

Methanotrophic bacteria can use methane (CH_4_), the primary component of natural gas and anaerobic digestion (AD)-derived biogas, as a sole carbon and energy source, presenting a promising biological route for atmospheric CH_4_ sequestration, bioremediation, and gas-to-liquid conversion for industrial applications (Kalyuzhnaya et al., 2015; Strong et al., 2015, 2016; Pieja et al., 2017). To this latter end, we have recently reported a biocatalytic route for methane conversion to lipid fuel intermediates and platform chemicals, as well as metabolic engineering strategies to enhance carbon conversion efficiency of biological gas-to-liquid conversion processes (Henard et al., 2016, 2017). Additional recent reports have demonstrated methane bioconversion to diverse product suites, including single cell protein, methanol, carboxylic acids, polyhydroxybutyrate, and 2,3-butanediol (Bothe et al., 2002; Hwang et al., 2014; Cal et al., 2016; Myung et al., 2016; Garg et al., 2018; Nguyen et al., 2018), further underscoring the potential power of methanotrophic bioconversion strategies.

Aerobic methanotrophs are ubiquitous in nature and serve as a primary environmental CH_4_ sink, significantly contributing to the global biogeochemical carbon cycle (Anthony, 1982). An array of methanotrophic bacteria have been isolated in pure culture and primarily belong to the diverse classes of gamma- and alphaproteobacterial (Hanson and Hanson, 1996). The gammaproteobacteria *Methylococcus capsulatus* Bath and alphaproteobacteria *Methylosinus trichosporium* OB3b have served as models for understanding the fundamentals of methanotrophy and have defined two primary pathways for CH_4_ assimilation in these organisms, the ribulose monophosphate pathway (RuMP) and the serine cycle, respectively. With a resurgent interest in applied methanotrophy (Conrado and Gonzalez, 2014; Haynes and Gonzalez, 2014; Strong et al., 2015; Clomburg et al., 2017), several novel methanotrophs have recently been isolated and their genomes sequenced, providing further insight into CH_4_ metabolism and the development of genome scale models (Boden et al., 2011; Khmelenina et al., 2013; Kits et al., 2013; Hamilton et al., 2015; Flynn et al., 2016; Akberdin et al., 2018).

Among the most promising of these recently isolated methanotrophs are the gammaproteobacterial, haloalkaliphilic members of the genus *Methylomicrobium*, including *Methylomicrobium alcaliphilum* 20Z^R^ and *Methylomicrobium buryatense* 5G(B1), which have established genetic tools and genome scale models (Ojala et al., 2011; Gilman et al., 2015; la Torre et al., 2015; Puri et al., 2015; Henard et al., 2016; Yan et al., 2016; Akberdin et al., 2018). Several methanotrophic strains possess unique characteristics for biotechnological deployment, including differential growth rates, cultivation parameters, flux to metabolic intermediates, and end-product tolerance. However, strain selection for industrial applications is not always obvious; while some basic considerations can be applied to all biological CH_4_ oxidation processes, the selection of a microbial catalyst is influenced by the type of application to be developed, including substrate source, product selection, and ultimately, the overall process economics of the technology (Kalyuzhnaya, 2016). Additionally, though bioconversion parameters are well-defined for pure CH_4_ in the above-described strains, the potential for methanotrophic cultivation and bioproduction on renewable, AD-derived biogas remains to be fully evaluated, limiting adoption, and impact as a core gas-to-liquid technology.

Biogas derived from AD of waste stream sources such as municipal solid waste operations, biorefineries, and agricultural operations, offers a versatile renewable energy source. At present, biogas is primarily used to produce combined heat and power (CHP). Alternatively, AD biogas can be scrubbed for conversion to biomethane that can, in turn, be utilized as a renewable option in natural gas applications. Total domestic methane potential from landfill material, animal manure, wastewater, and organic waste (food waste) is estimated to be >400 TBtu (Department of Energy, 2017). Additionally, biogas generated from AD of lignocellulosic biomass resources is estimated to offer >4 quadrillion Btu potential energy (Department of Energy, 2017). This energy potential could displace nearly half of current domestic natural gas consumption in the electric power sector and all current natural gas consumption in the transportation sector (Department of Energy, 2017). Despite the promise of biogas as a high-volume, renewable energy source and natural gas replacement, its gaseous state prevents facile integration with extant transportation and industrial infrastructure. Additionally, biogas composition varies significantly depending upon input feedstock, but it is typically comprised of 40–65% CH_4_, 30–40% carbon dioxide (CO_2_), and gaseous impurities, including hydrogen sulfide (H_2_S), ammonia, and siloxanes (Hosseini and Wahid, 2014).

In this study we explored the applicability of phylogenetically diverse methanotrophic bacteria for AD biogas utilization and conversion. We tested six variable sources of biogas derived from AD of energy crops and derivatives thereof, conducting comparative growth analyses of three representative methanotrophic cultures, *M. capsulatus* Bath, *M. trichosporium* OB3b, and *M. alcaliphilum* 20Z^R^. The impact of the various biogas streams on cellular metabolism was further investigated in *M. alcaliphilum* 20Z^R^ using global metabolomics analysis. Lastly, we demonstrated biogas conversion to lactate at the highest reported specific productivity to date by a rationally engineered *M. alcaliphilum* 20Z^R^ pyruvate dehydrogenase mutant.

## Materials and methods

### Bacterial strains and cultivation

*Methylomicrobium alcaliphilum* 20Z^R^ (Akberdin et al., 2018), *Methylosinus trichosporium* OB3b, and *Methylococcus capsulatus* Bath were cultivated in either nitrate mineral salts (NMS) medium (Bath and OB3b) or NMS medium supplemented with 3% NaCl and carbonate buffer as previously described (Whittenbury et al., 1970; Akberdin et al., 2018). To determine optimal biogas concentration and biogas effects on growth, cultures were grown in 250 ml vials containing 50 ml of growth medium. After inoculation at a starting density of OD_600_ = 0.10, the vials were crimped with butyl (gray or red) stoppers to create gas-tight seals. Increasing concentrations of mock biogas (~60% CH_4_/40% CO_2_) or pure CH_4_ was added to the headspace to determine the optimal biogas concentration for growth. Biogas samples BG1-6 were added to the headspace [33% biogas (~20% CH_4_) in air] of serum vials to evaluate their effects on methanotrophic growth. Cultures containing pure CH_4_ were supplemented with nitrogen to equilibrate the volume of gas added to the corresponding biogas serum vial. Cultures were incubated at 30°C (20Z^R^ and OB3b) or 37°C (Bath) with orbital shaking at 200 rpm, and bacterial growth was measured spectrophotometrically. A second series of parallel cultures were set up for headspace composition (CH_4_, N_2_, O_2_, CO_2_, and CO) analyses and biomass yield. At each timepoint, samples of the headspace (1 ml) were injected into an SRI GC system for gas chromatography analyses. Gas consumption data were collected at the beginning and completion of each experiment. The concentrations were estimated using standard gas mixtures (Scotty Analyzed gases, Supelco Analytical, Sigma-Aldrich). Dry Cell Weight (DCW) was either measured directly after freeze-drying or estimated from the final OD of the cell culture using the following equation: DCW = OD ^*^ (0.35 ± 0.04 g/L) (Akberdin et al., 2018). Biomass yield data (Y_CH4_) were calculated using dry cell weight and consumed substrate data and represented as g biomass produced per g CH_4_ consumed.

### Anaerobic digestion and biogas generation

Various feedstock substrates were received from Idaho National Laboratory, the Ohio Soybean Council, Aemetis, and University of Illinois, Urbana-Champaign. Continuous digestions were performed in six lab-scale digesters operating at 14-L net volume per digester, which were inoculated with digester content obtained from East Bay Municipal Utility District and a local dairy (Straus, CA). Reactors were stabilized to yield equal base load gas production and began continuous operation at a loading rate ramped up to 2 kg organic dry matter per cubic meter reactor volume per day with a target hydraulic retention (HRT) of 21–28 days and run time of 2.5 HRT, monitoring for gas flow, pH and gas composition. We characterized the material composition, the theoretical biogas and CH_4_ yield per the models of Buswell and Baserga (Achinas and Euverink, 2016), as well as the batch yield per VDI 4630 for soybean residues, corn stover, miscanthus, switchgrass, sorghum, bagasse, and two different ethanol stillage streams. In the continuous operation, we measured and quantified gas composition using gas chromatography compared to known standards.

### Metabolite profiling

Intracellular metabolites were analyzed by Metabolon, Inc. (Durham, NC) from *M. alcaliphilum* 20Z^R^ cultured in serum vials with the headspace supplemented with 33% biogas (~20% CH_4_) in air as described above_._ Cells were collected by centrifugation when cultures reached OD_600_ = 0.6–0.7 and frozen in liquid nitrogen and stored at −80°C prior to shipping to Metabolon. Metabolomic profiles were collected and processed as previously described (Henard et al., 2017; Akberdin et al., 2018). Changes in cell samples grown on biogas were compared to cell cultures grown on equivalent concentrations of CH_4_. Welch's two-sample *t*-tests and Principal Component Analysis (PCA) were used to analyze the data. For all analyses, following normalization to protein measured by Bradford, missing values, if any, were imputed with the observed minimum for that particular compound. The statistical analyses were performed on natural log-transformed data and were considered significant if *p* < 0.05.

### Mutant construction

Strains, plasmids, and primers used for amplification of upstream and downstream regions for construction of the pyruvate dehydrogenase (*pdh*) knockout are shown in Table S1. Genomic fragments, ~600-bp of sequences flanking the dihydrolipoamide acetyltransferase subunit of the pyruvate dehydrogenase complex (MALCv4_1358) gene, were amplified by PCR, and cloned into pCM184::Gm^R^ plasmid at AatII/NcoI (upstream region) and SacI/SacII (downstream region) sites. The resulting plasmid was introduced to the 20Z^R^ strain by biparental conjugation as described previously (Puri et al., 2015). After mating, gentamycin-resistant clones were selected on medium supplemented with acetate (5 mM), rifampicin (50 μg/mL), and gentamycin (30 μg/mL) to counter-select against *E. coli*. Then, the resulting colonies were PCR-genotyped for the absence of MALCv4_1358 gene followed by sequence verification.

### Continuous gas fermentation

Fifty milliliter cultures of wild-type and Δ*pdh::*Gm^R^ *M. alcaliphilum* 20Z^R^ were grown in 150 mL bubble columns with a continuous gas flow (20% CH_4_ or 33% mock biogas in air, 1 vvm). At indicated intervals, pH was determined, growth was measured spectrophotometrically, and a 1 mL sample was taken for HPLC analysis. After 96 h, bacteria were pelleted and freeze-dried to determine dry cell weight. HPLC was used to detect lactate in the culture supernatants. The culture supernatant was filtered using a 0.2 μm syringe filter and then a 0.1 mL injection was separated using a model 1260 HPLC (Agilent, Santa Clara, CA) and a cation H HPx-87H column (Bio-Rad). A 0.6 mL/min flow rate at 55°C with 0.01 N sulfuric acid as the mobile phase was used. DAD detection was measured at 220 nm and referenced at 360 nm, and metabolite concentrations were calculated by regression analysis compared to known standards. The identity of lactate was also confirmed by NMR analysis.

## Results and discussion

### Anaerobic digestion of crop residues

AD substrates were loaded into six 14-L lab scale continuously operating digesters. Steady state off-gas analyses, including H_2_S and other contaminants, are shown in Table 1. The CH_4_ and CO_2_ content were found to be consistently between 48–67% and 33–52%, respectively. The trace gases ethane, propane, n-butane, and n-propane were detected at < 250 ppm in all biogas streams. H_2_S content varied significantly between 80 and 14,000 ppm, with the highest H_2_S content found in the BG3 derived from a feedstock that is much higher in protein content compared to the other AD substrates, which increases gaseous sulfur components but also increases the CH_4_ content of the gas stream (Achinas and Euverink, 2016).

**Table 1:** Composition of tested biogas samples.

**Gas sample ID**	**Substrate**	**CH_4_ (%)**	**CO_2_ (%)**	**H_2_S (ppm)**	**Trace (< 250 ppm)**
BG1	Sorghum	49	50	2,100	CO
BG2	Corn stover	51	49	350	CO, Carbonyl sulfide (COS)
BG3	PEI syrup	67	33	14,000	Hexane, CO, COS
BG4	Bagasse	48	52	200	CO, COS
BG5	Corn distiller's solids syrup	63	37	13,000	CO, COS, C-5, C-6
BG6	Miscanthus	52	49	80	CO

### Methanotroph culture parameters on AD-derived biogas

**Figure 1:**
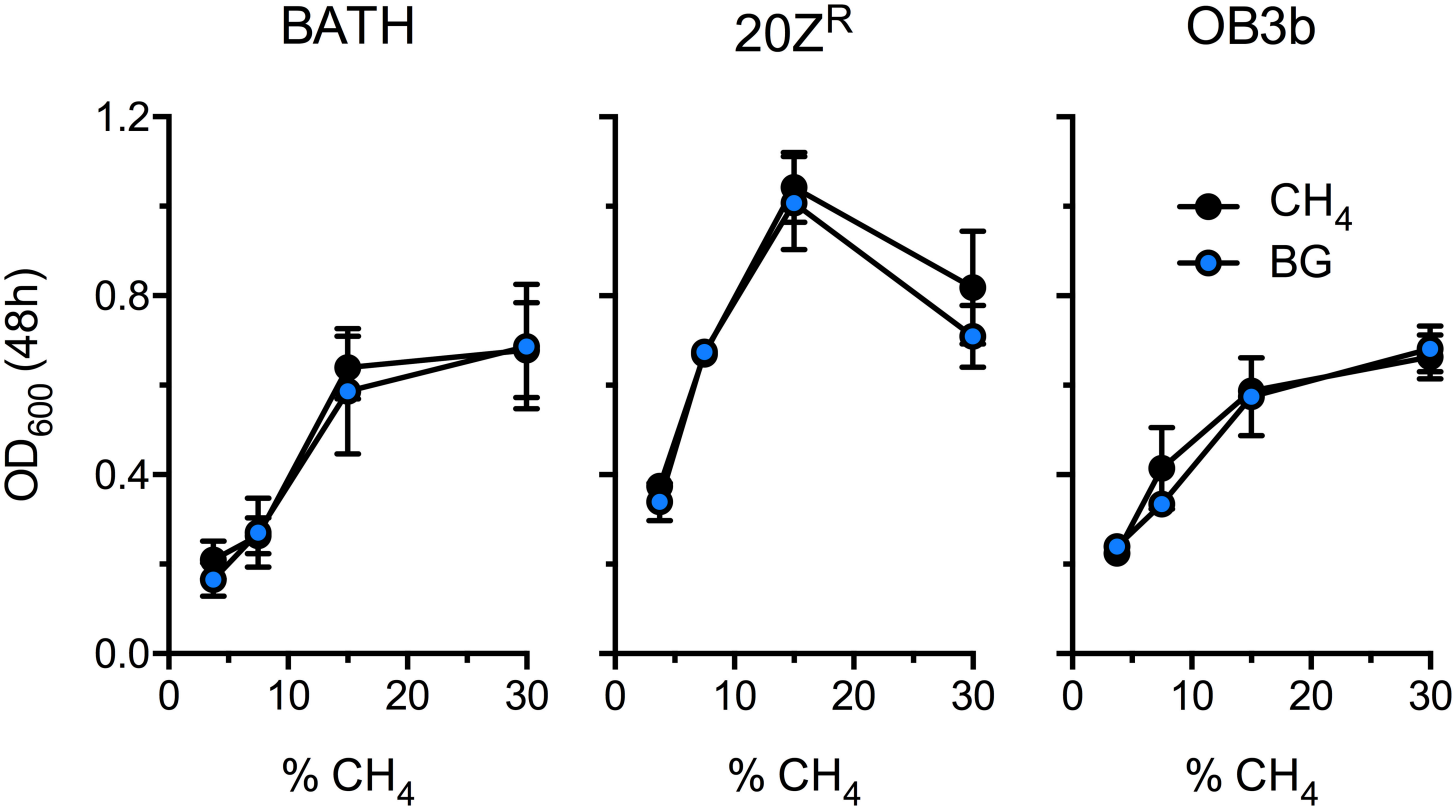
Determination of the optimal biogas dilution for methanotroph cultivation. *M. capsulatus* Bath (BATH), *M. alciliphilum* 20Z^R^ (20Z^R^), and *M. trichosporium* OB3b (OB3b) were cultivated in 250 mL serum vials with increasing concentrations of pure CH_4_ (black) or biogas (blue) added to the headspace. The data represent the average OD_600_ ± S.D. from four independent observations.

Methanotrophic bacteria require oxygen to activate CH_4_. Thus, AD-derived biogas will require mixing with air or pure oxygen before delivery to a methanotrophic biocatalyst. Further, CO_2_, H_2_S, and other biogas components have the potential to negatively affect bacterial growth. To determine the optimal biogas:air ratio we compared the growth of three diverse methanotrophs with mock biogas (60% CH_4_, 40% CO_2_, 0.01% H_2_S) or pure CH_4_ at varying concentrations ranging from 3.5% to 30% CH_4_. The growth of all strains positively correlated to CH_4_ concentration in the headspace, presumably due to increased gas availability in the medium, with strains cultivated on 15–30% CH_4_ displaying optimal growth (Figure 1). Further, we observed no difference in growth between cultures supplied with biogas or pure CH_4_, suggesting that high CO_2_ levels (20% v/v) does not negatively affect these organisms under these growth conditions (Figure 1). Interestingly, cultures exhibited optimal growth with a much higher CH_4_:O_2_ ratio (3:1) than is conventionally used for methanotroph cultivation (1.25:1), indicating that methanotrophic growth is not oxygen limited under our experimental conditions.

We next evaluated methanotrophic growth on the six AD-derived biogas streams (Table 2). The growth of *M. alcaliphilum* 20Z^R^ was inhibited by biogas originating from miscanthus silages (BG6); however, the strain grew comparable to pure CH_4_ on all other biogases. *M. capsulatus* Bath growth was inhibited by both sorghum (BG1)-and miscanthus (BG6)-derived biogases, while *M. trichosporium* OB3b was only slightly inhibited by sorghum (BG1)-derived biogas. Interestingly, OB3b displayed increased growth kinetics in PEI syrup (BG3)- and CDS syrup (BG5)-derived biogas streams. Collectively, all three cultures displayed growth capacity on biogas with only minor alterations in biomass yield from CH_4_. However, some differences in biomass yield and doubling time were observed between biogas streams, underscoring that biogas composition can dictate the methanotrophic biocatalyst most appropriate for its conversion.

**Table 2:** Microbial cultures parameters on varied biogas streams.

**Carbon source**	***M. alcaliphilum*** **20Z**^**R**^		***M. capsulatus*** **Bath**		***M. trichosporium*** **OB3b**	
	***Y_*B*_***	***T_*d*_***	***Y_*B*_***	***T_*d*_***	***Y_*B*_***	***T_*d*_***
CH_4_	1.03	5.84	0.85	6.72	0.78	10.9
BG1	0.95	7.38	0.81	13.0	0.69	14.9
BG2	0.93	7.53	0.86	7.74	0.68	12.7
BG3	0.96	6.03	0.87	6.95	0.73	8.63
BG4	0.95	7.49	0.94	7.98	0.73	13.0
BG5	0.96	6.46	0.93	7.00	0.70	9.50
BG6	0.60	11.0	0.40	12.1	0.71	12.7

*Y_B_, biomass yield (g DCW/g CH_4_); T_d_, doubling time (h). The data represent the mean 2–3 independent observations*.

### Biogas-induced metabolic alterations

In order to better understand the impacts of raw biogas feedstock on the growth of methanotrophic bacteria, a series of metabolomic experiments were carried out. Since *M. alcaliphilum* 20Z^R^ was superior to the other cultures with respect to growth and efficiency of biogas conversion, this strain was further evaluated for biochemical profiling. A summary of metabolites significantly altered during cultivation on biogas is shown in Table S2. As expected from the growth inhibition data, biogas derived from miscanthus (BG6) led to the most significant metabolite alterations. A PCA plot revealed that the treatment-related variation between groups was only slightly greater than the biological noise within groups (Figure S1). This suggests that the treatments did not cause profound perturbations in metabolism relative to the control condition. Indeed, metabolites of core metabolic pathways, including glycolytic, tricarboxylic acid, and pentose phosphate/ribulose 5 phosphate pathway metabolites were similar between CH_4_ and the six AD-derived biogas samples (Table S2).

**Figure 2:**
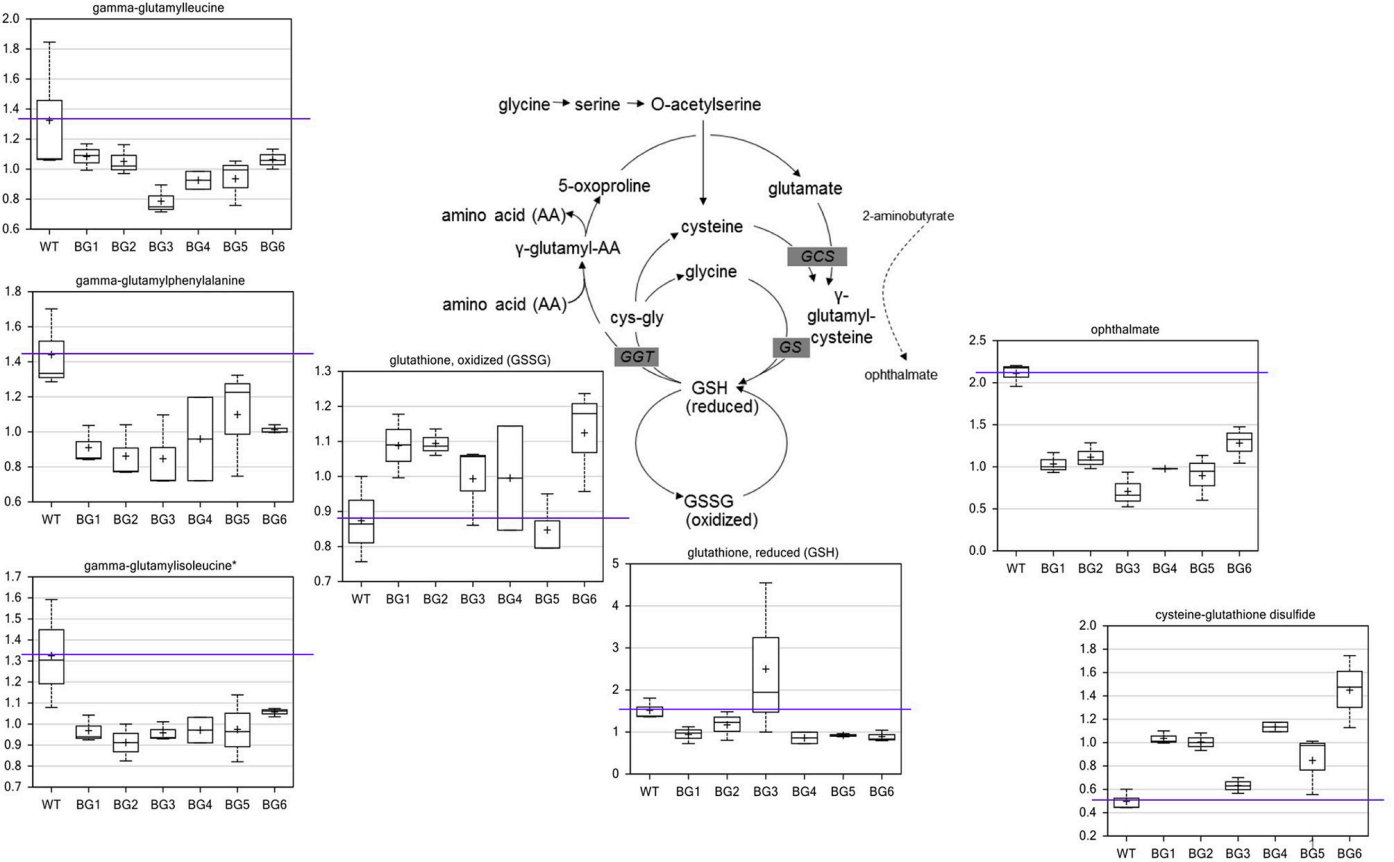
Biogas-induced alterations in the redox state of glutathione and biosynthetic pathway intermediates. Metabolomic analysis of compounds associated with glutathione metabolism significantly altered during cultivation on variable biogas streams. The data represent the average ± S.D. of three independent biological samples.

When compared to the pure CH_4_-grown controls, several metabolite alterations pointed to a general effect on redox state related to biogas feeding. The most significant difference in metabolites of biogas-grown samples were in the glutathione biosynthetic pathway (Figure 2). In five of the six treatments, the levels of oxidized glutathione (GSSG) were higher while reduced glutathione (GSH) was generally lower. The oxidation product of glutathione and cysteine, cysteine-glutathione disulfide, was also higher in all biogas-grown cells. The data indicate that the pure CH_4_-fed cells contained more favorable levels of reduced glutathione, as well as a greater capacity to produce the compound. Consistent with this, several *gamma*-glutamyl amino acids, which are co-products of glutathione recycling, were also lower in all the treatment groups. The main biosynthetic enzymes for GSH production, *gamma*-glutamylcysteine synthetase (GCS) and glutathione synthetase (GS), can also generate a side product, ophthalmate, when GCS incorporates 2-aminobutyrate instead of cysteine and then GS adds the glycine to this non-specific intermediate. We observed a conserved decrease in ophthalmate in all biogas-cultivated samples. Interestingly, ophthalmate has previously only been found in mammals and the cyanobacteria *Synechocystis* (Soga et al., 2006; Narainsamy et al., 2016). It is unknown whether ergothioneine and/or ophthalmate function as effective antioxidants in *M. alcaliphilum* 20Z^R^, but our data suggest they are, along with GSH, associated with a general antioxidant response. However, there are very few reports detailing antioxidant responses or glutathione-mediated reactions in methanotrophic bacteria. Thus, the linked pathways could be targeted for further investigation for improving biogas utilization.

Compounds in other pathways also supported that biogas-treated cells were more oxidized or experiencing higher relative oxidative stress. Elevation of sulfate and the histidine derivative ergothioneine was observed in all the biogas cultures (Table S2). Ergothioneine levels were roughly correlated with sulfate levels across the different biogas treatments. Also, various one-carbon derivatives of organic acids and amino acids (methylmalonate, methylsuccinate, *N-*formylmethionine, 2-methyserine) were higher in all the biogas treatments relative to the CH_4_ control. The metabolite measured as the most significantly altered during biogas cultivation was ribonate, the oxidation product of ribose (Table S2). This compound ranged from 10- to 20-fold higher than CH_4_-grown controls in all biogas cultivated samples. The enzyme ribose-1 dehydrogenase is NADP+ dependent (in some organisms) and can serve to provide NADPH-reducing equivalents to the system. Other compounds, such as UDP-glucuronate and pyridoxate, which are more highly oxidized versions of common metabolites, were also higher in all the biogas-grown cells. The oxidation of UDP-glucose to UDP-glucuronate by UDP-glucose dehydrogenase also produces reducing equivalents in the form of NADH. Taken together with the increased oxidized glutathione levels, these data support that components in biogas, CO_2_, and/or contaminants, alter the intracellular redox state of the methanotroph.

### Bioconversion of biogas to lactate in a continuous gas flow bioreactor

**Figure 3:**
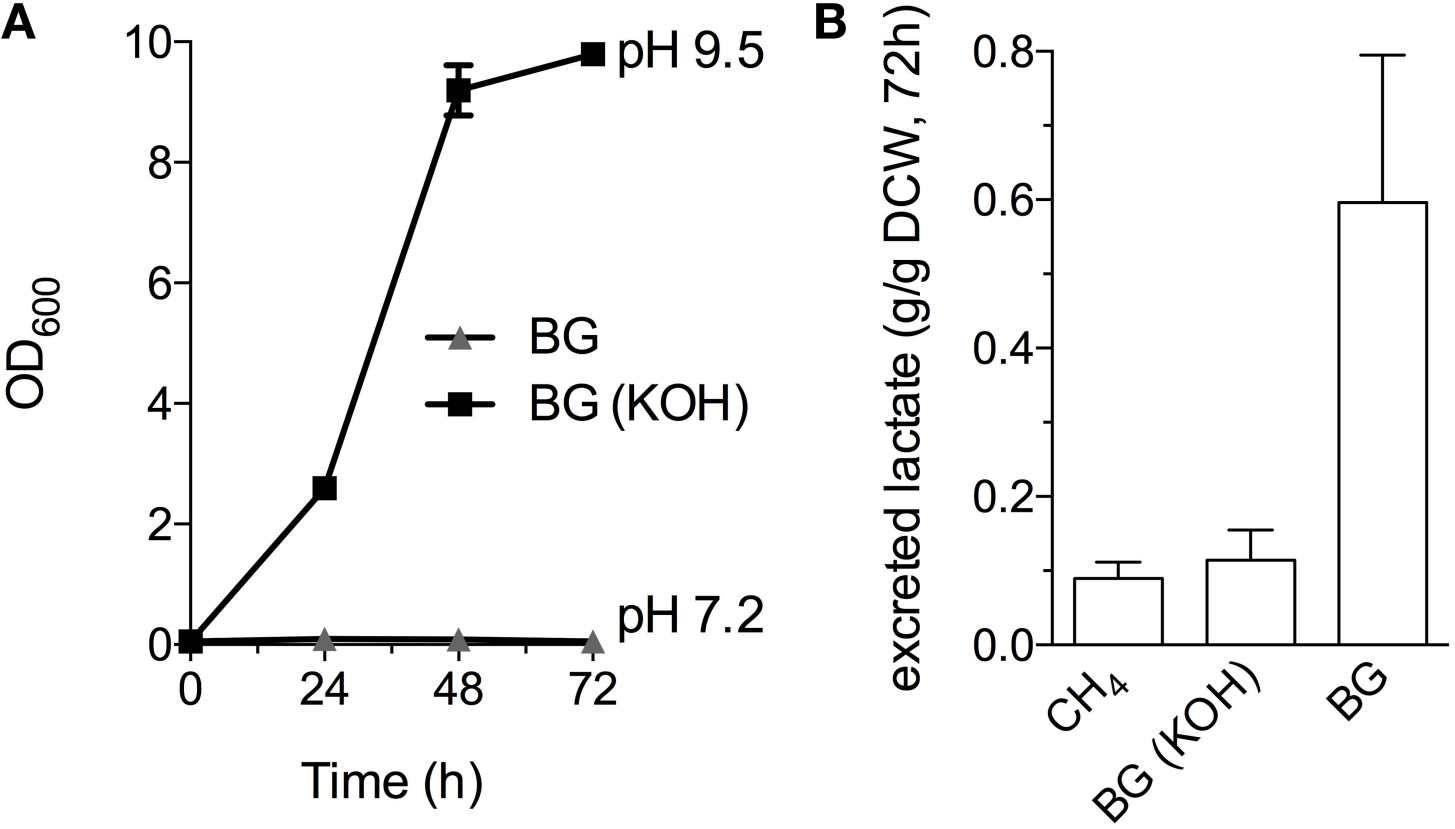
*M. alcaliphilum* 20Z^R^ cultivation on a continuous biogas stream. **(A)** Bacterial growth with continuous supply of 33% biogas in air (20% CH_4_, 13% CO_2_, 1 vvm) with (black square) or without (gray triangle) the addition of 0.4N potassium hydroxide (KOH). **(B)** Lactate excreted into the culture medium by *M. alciliphilum* supplied with CH_4_ or biogas (with or without KOH in the medium) detected by HPLC and normalized to dry cell weight (DCW).The data represent the average ± S.D. from three independent observations.

**Figure 4:**
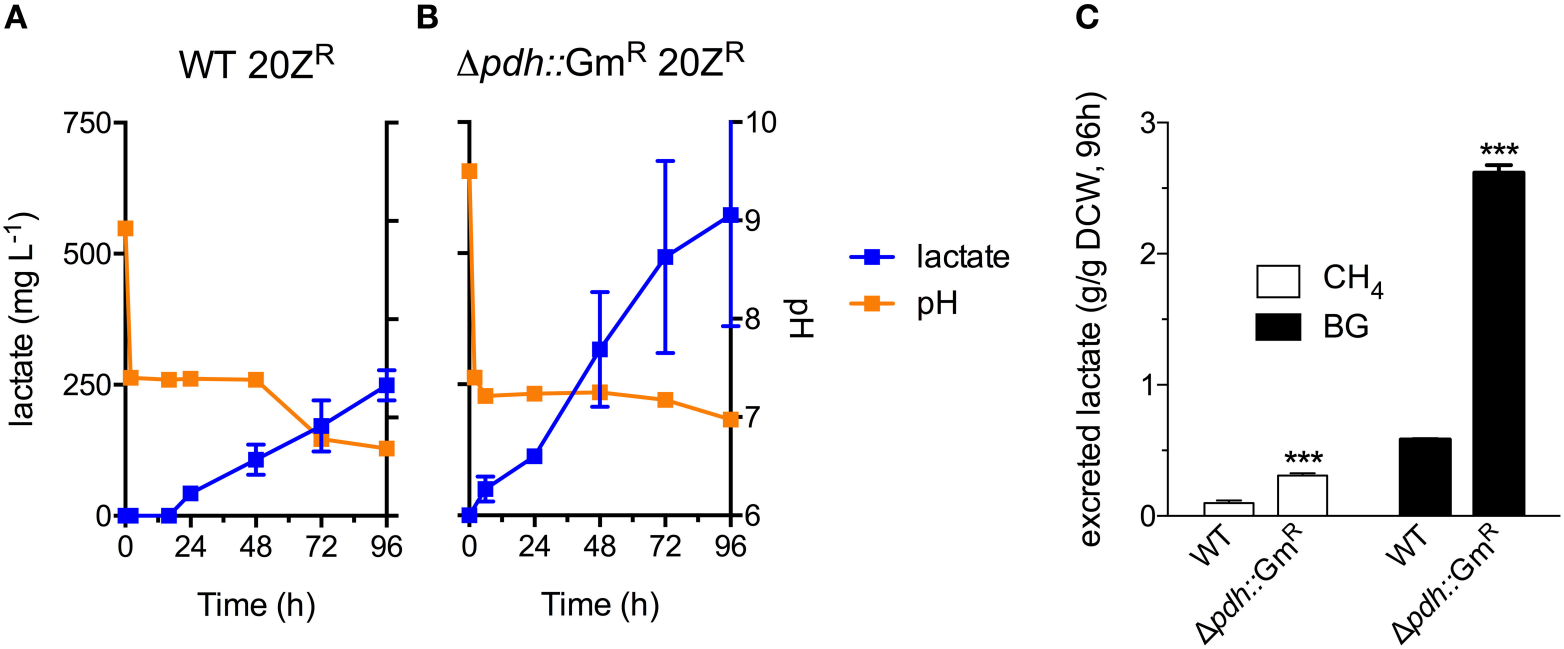
A *M. alcaliphilum* 20Z^R^ pyruvate dehydrogenase mutant exhibits increased flux to lactate. Excreted lactate (blue square), and culture pH (orange square) of wild-type (WT, **A**) and pyruvate dehydrogenase mutant (Δ*pdh*::Gm^R^, **B**) *M. alcaliphilum* 20Z^R^ during cultivation with continuous supply of 33% biogas in air (20% CH_4_, 13% CO_2_, 1 vvm) **(C)** Lactate flux from pure CH_4_ (white bars) or biogas (black bars) in WT and Δ*pdh*::Gm^R^ *M. alcaliphilum* 20Z^R^ based on dry cell weight (DCW). The data represent the average ± S.D. from 2 to 4 independent observations. ***p < 0.001 compared to wild-type controls.

Industrial processes employing methanotrophic bacteria currently operate using a continuous natural gas supply, and future industrial bioconversion of AD-derived biogas will likely require a continuous gas fermentation mode. Thus, we evaluated *M. alcaliphilum* 20Z^R^ growth in a mid-throughput gas fermentation reactor supplied with 33% biogas in air (20% CH_4_, 13% CO_2_) at 1 volume of gas per volume of liquid per minute. Surprisingly, we observed no bacterial growth under continuous biogas supply, potentially due to carbonic acid production as indicated by a significant drop in the pH of the culture medium (Figure 3A and Figure S2). Bacterial growth was restored by the addition of KOH that raised the pH of the medium to pH 9.5 (Figure 3A), suggesting that H_2_S or other biogas components not affecting culture pH did not affect bacterial growth. HPLC analysis of the culture medium showed that lactate was the primary organic acid secreted during cultivation under continuous gas supply, with equivalent lactate (between 80 and 120 mg/L) detected from cultures grown with pure CH_4_ or biogas buffered with KOH after 72 h of cultivation (Figure 3B). Interestingly, we detected increased lactate production (between 220 and 280 mg/L) from cultures with biogas-inhibited growth during the same cultivation timeframe (Figure 3B). Lactate is predicted to be synthesized by *M. alcaliphilum* 20Z^R^ via the conversion of pyruvate to lactate by a lactate dehydrogenase (LDH, MALCv4_0534). We hypothesized that flux to lactate could be improved by removing pyruvate conversion to acetyl-CoA, the primary carbon flux during active growth of gammaproteobacterial methanotrophs mediated by the pyruvate dehydrogenase (Kalyuzhnaya et al., 2013; Akberdin et al., 2018). Indeed, we observed a significant increase in both lactate titer (2-3 fold) and specific productivity (four-fold) in a pyruvate dehydrogenase mutant compared to wild-type *M. alcaliphilum* 20Z^R^ when cultured with continuous CH_4_ or biogas feed (Figure 4). These data suggest that this promising biocatalyst increases lactate biosynthesis and excretion in response to the low pH induced by biogas-derived carbonic acid, representing a promising fermentation configuration for organic acid production. Further, this represents the highest reported lactate specific productivity (0.027 g lactate/gDWC/h) in a methanotroph expressing its native LDH, significantly improved compared to our previous demonstrations of CH_4_ bioconversion to lactate (Henard et al., 2016). In addition to the deletion of the pyruvate dehydrogenase, overexpression of the native *M. alcaliphilum* 20Z^R^ LDH or a heterologous LDH with known minimal negative feedback regulation like the *Lactobacillus helveticus* LDH, is a rational metabolic engineering target for increased pyruvate conversion to lactate (Henard et al., 2016; Garg et al., 2018).

## Conclusion

Methanotrophic bacteria have recently gained intensified biotechnological interest due to their capacity to use methane as a sole carbon and energy source, in turn presenting a promising gas-to-liquid bioconversion pathway. Though numerous technology-to-market hurdles remain, these efforts serve as proof-of-concept for microbial conversion of AD-derived biogas, notably presenting a modular, up-, and down-scalable, and highly selective route to fuel and chemical intermediates.

Our data indicate that cultivation of diverse methanotrophic bacteria is feasible on biogas derived from energy crops and residues, despite containing high levels of toxic contaminants. Metabolite profiling of *M. alcaliphilum* 20Z^R^ supports that biogas components alter the intracellular redox state of this organism, which can be leveraged to guide future metabolic engineering efforts in development of efficient biogas biocatalysts. Importantly, we demonstrated bioconversion of biogas to lactate by *M. alcaliphilum* 20Z^R^, and improved lactate specific productivity via rational strain engineering in this methanotroph. Lactate is a promising chemical precursor for the production of bioplastics (Abdel-Rahman et al., 2013; Eiteman and Ramalingam, 2015), and can also be used to generate an array of additional chemical building blocks, including acrylic acid, propylene glycol, and pentanol. These chemical intermediates, along with polymers and fuels that could be generated from biogas, offer a viable, renewable alternative to those generated from conventional carbohydrate feedstocks, which compete with food production. Biocatalysis of conventionally flared AD biogas has the added benefit of GHG reduction while also offering a means to concurrently liquefy and upgrade CH_4_, enabling its utilization in conventional transportation and industrial manufacturing infrastructure. Future integration of biogas biocatalysis into conventional AD and biorefinery infrastructure will provide insight into other opportunities for recycling and cost reductions, advancing a viable route to a greener bioeconomy.

## Author contributions

CH, MG, and MK designed experiments and analyzed the data. CH, TF, BY, SB, and DA performed experiments. CH, MK, DA, and MG wrote the manuscript. All authors approved the final version of the manuscript.

### Conflict of interest statement

The authors declare that the research was conducted in the absence of any commercial or financial relationships that could be construed as a potential conflict of interest.
