# Supplementary material for: Biogas Biocatalysis: Methanotrophic Bacterial Cultivation, Metabolite Profiling, and Bioconversion to Lactic Acid
Source: Front Microbiol. 2018 Oct 31;9:2610. doi: 10.3389/fmicb.2018.02610 (PMC6220066; doi:10.3389/fmicb.2018.02610)
Supplement: Supplementary file 1 [file Table_1.docx]

**Table S1.** Strains, plasmids, and primers used in this study

| **Strains** | **Description** | **References** |
| --- | --- | --- |
| 20Z^R^ | Rifimycin resistant derivative of *Methylomicrobium alcaliphilum* 20Z | (Akberdin et al., 2018) |
| 20Z^R^  Δ*pdh::*Gm^R^ | 20Z^R^ strain lacking pyruvate dehydrogenase complex dihydrolipoamide acetyltransferase **(**MALCv4_1358) gene, gentamicin resistant | This study |
| **Plasmids** |  | This study |
| pCM184 | Broad Host Range vector. Vector backbone pAYC61 | (Marx and Lidstrom, 2002) |
| pCM184::Gm^R^ | pCM184 backbone, kanamycin cassette replaced with gentamicin cassette | This study |
| pSB2 | pCM184::Gm^R^ with MALCv4_1358 up/down flanks | This study |
| **Primers** |  |  |
| Pdh-up/F-AatII | gagacgtcGGAAGAACGACGCTCAACGGCG | This study |
| Pdh-up/R-NcoI | gaccatGGCGCCGATAGGTAAGGACG | This study |
| Pdh-dw/R-SacI | gagagctcGGTCGAATCCCACAGACGC | This study |
| Pdh-Dw/F-SacII | gaccgcgGCGCCTAGACTCAAACGTCGAG | This study |

**Table S2.** Summary of compounds significantly altered in cell cultures grown on biogas compared to cell cultures grown on pure methane. *see accompanying spreadsheet file


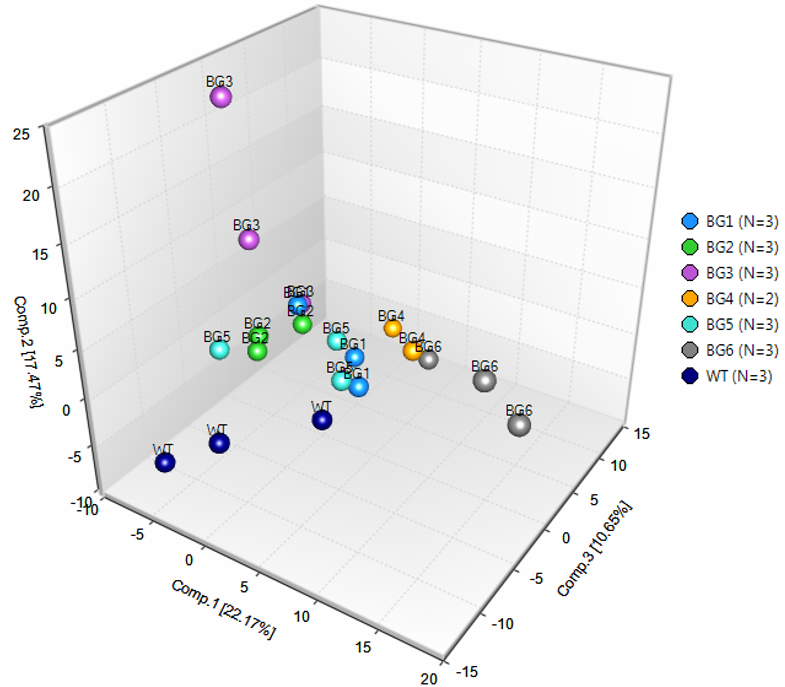


**Figure S1.**  PCA of biogas cultivated samples.


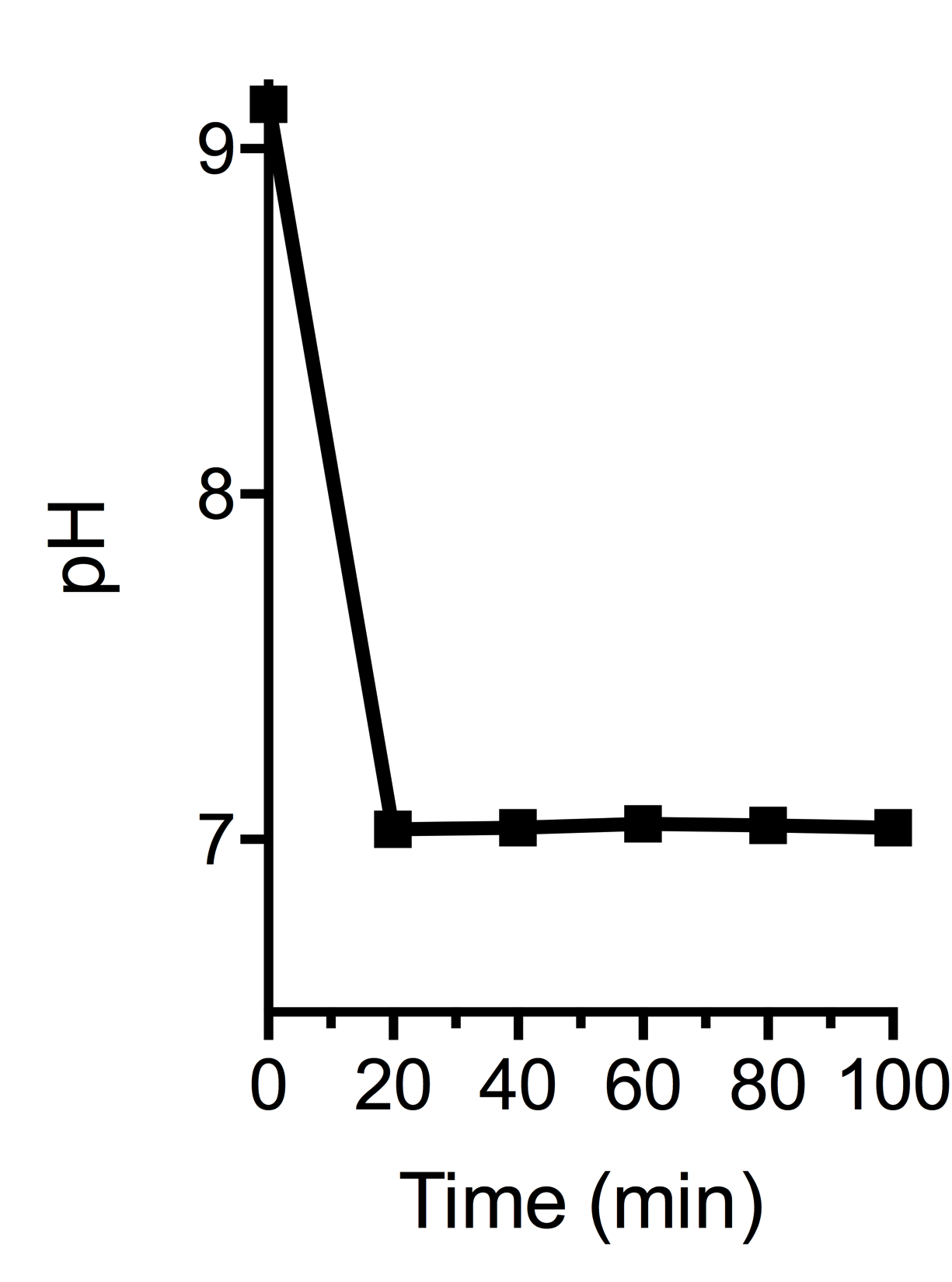


**Figure S2.** pH of abiotic growth medium with continuous supply of 33% mock biogas (20% CH_4_, 13% CO_2_, 1vvm). The data represent the mean pH ± S.D. of two independent observations.

REFERENCES

Akberdin, I. R., Thompson, M., Hamilton, R., Desai, N., Alexander, D., Henard, C. A., et al. (2018). Methane utilization in Methylomicrobium alcaliphilum 20Z R : a systems approach. *Sci Rep* 8, 2512. doi:10.1038/s41598-018-20574-z.

Marx, C. J., and Lidstrom, M. E. (2002). Broad-host-range cre-lox system for antibiotic marker recycling in gram-negative bacteria. *Biotechniques*.
